# The forensic assessment of urgent involuntary psychiatric admissions in Barcelona (Spain)

**DOI:** 10.1192/j.eurpsy.2025.396

**Published:** 2025-08-26

**Authors:** J. Casanovas, A. Martínez, C. Fuentes, M. Vilella, E. Barbería, A. Xifró

**Affiliations:** 1 Institut de Medicina Legal i Ciències Forenses de Catalunya, Barcelona; 2 Universitat Rovira i Virgili, Reus; 3 Universitat de Barcelona, Barcelona, Spain

## Abstract

**Introduction:**

In Spain, involuntary admission due to mental disorder requires judicial authorization, which can be prior or, in cases of urgency, immediately subsequent. The courts routinely request an independent medical expert opinion in these cases.

**Objectives:**

Our aim was to determine the current results of these evaluations and the characteristics of the patients assessed.

**Methods:**

Retrospective study from January 1 to June 30, 2023 in the city of Barcelona. The source of information were the case records at the Institute of Legal Medicine and Forensic Sciences of Catalonia. The inclusion criteria were: psychiatric involuntary admission, urgent, and assessment by the forensic doctor assigned to the courts. Sociodemographic, clinical and forensic data were collected.

**Results:**

In the 181 days between January 1 and June 30, 2023, 1,151 forensic medical assessments of urgent hospitalizations were recorded in Barcelona (on average 6.4 per day). Of these, 849 (73.8%) were included. In all of them, the forensic medical report concluded that hospitalization was rightly indicated. The admissions were carried out in 14 different health centers. The psychiatric acute inpatient wards of the city’s main general hospitals received the vast majority of cases. The patients were mostly men (n=483; 56.9%). The average age was 38.2 years (s.d. 16.99), with no differences between sexes. 13.5% (n=115) were minors, with female predominance (n=79; 68.7%). 6.9% (n=59) were 65 years of age or older, also with a female predominance (n=39; 66.1%). In minors, affective disorders (n=37; 32.7%) or eating behavior disorders (n=32; 28.3%) stood out, while in the group from 18 to 65 years of age the main diagnostic group were psychotic disorders (n =491; 73.1%). The latter were also the majority after 65 years of age (n=35; 59.3%), followed by affective disorders (n=16; 27.1%). Globally, psychotic disorders were the most frequent diagnostic group for both men (n=346; 71,9%) and women (n=188; 51,8%). Affective or eating behaviour disorders accounted for 35,8% of women (n=130) and 18,3% of men (n=88).

**Conclusions:**

The forensic assessment of the medical indication of urgent involuntary psychiatric hospitalizations coincides with the clinical assessment. Patients with this measure show differential characteristics according to age in terms of gender and diagnosis. More men are involuntarily admitted than women for most of the adult stage, while the reverse is true at both extremes of the lifespan. Also, involuntarily admitted women show a higher frequency of mood or eating behaviour disorders than men.

**Disclosure of Interest:**

None Declared

